# Risk factors for the development of striae gravidarum

**DOI:** 10.1016/j.ajog.2006.08.044

**Published:** 2007-01

**Authors:** Hibah Osman, Nelly Rubeiz, Hala Tamim, Anwar H. Nassar

**Affiliations:** 1 Department of Health Behavior and Education, Faculty of Health Sciences, American University of Beirut Medical Center, Beirut, Lebanon; 2 Department of Dermatology, American University of Beirut Medical Center, Beirut, Lebanon; 3 Department of Epidemiology and Biostatistics, American University of Beirut Medical Center, Beirut, Lebanon; 4 Faculty of Health Sciences, and Department of Obstetrics and Gynecology, American University of Beirut Medical Center, Beirut, Lebanon.

**Keywords:** estrogen, pregnancy, relaxin, striae gravidarum

## Abstract

**Objective:**

The purpose of this study was to identify risk factors associated with striae gravidarum (SG).

**Study design:**

A cross-sectional study of 112 primiparous women delivering at a private teaching hospital was conducted. Participants were assessed during the immediate postpartum period for evidence of SG. Presence and severity of SG were compared to characteristics of women using *t* tests and Chi-square tests.

**Results:**

Sixty percent of the study participants had developed SG. Women who developed SG were significantly younger (26.5 ± 4.5 vs 30.5 ± 4.6; *P* < .001) and had gained significantly more weight during pregnancy (15.6 ± 3.9 vs 38.4 kg ± 2.7; *P* < .001). Birthweight (BW), gestational age at delivery, and family history of SG were associated with moderate/severe SG.

**Conclusion:**

Maternal age and weight gain during pregnancy are associated with SG. BW, family history of SG, and gestational age at delivery are associated with moderate/severe SG.

Striae distensae or “stretch marks,” referred to as striae gravidarum (SG) when they occur in pregnancy, are a common skin problem of considerable cosmetic concern to many patients. They are characterized clinically by linear bands that are initially erythematous to violaceous and gradually fade to become skin colored or hypopigmented atrophic lines that may be thin or wide. SG occur on the abdomen, breasts, buttocks, hips, and thighs and usually develop after the 24th week of gestation.^1^

The cause of SG remains unknown but clearly relates to changes in the structures that provide the skin with its tensile strength and elasticity. Mechanical stretching of the skin in association with hormonal factors has been implicated in the pathogenesis.^2-5^ It has been postulated that some hormones, like estrogen, relaxin, and adrenocortical hormones, decrease the adhesiveness between collagen fibers and increase ground substance, which results in the formation of striae in areas of stretching.^6^ Striae may form due to structural connective tissue changes that include realignment and reduced elastin and fibrillin in the dermis.^7^ However, some studies have shown that although SG tend to occur in areas of maximum skin stretching, there is no correlation between the degree of striae formation and the extent of body size enlargement during pregnancy.^8^ A recent study has found a correlation between the presence of striae and pelvic relaxation, a condition associated with decreased collagen content.^9^ This study was limited in that it used self-reported data, and physical exams were not performed.

The data on prevalence of SG and the risk factors associated with their development are scant and often contradictory. It is estimated that up to 90% of pregnant women develop SG^3^; however, some authors report the prevalence to be as low as 50%.^10^ Proposed risk factors for the development of SG include family history, race, skin type, birthweight (BW), baseline body mass index (BMI), age, weight gain, and poor nutrition; however, most of these have not been substantiated.^1,3-5^

In preparation for a clinical trial on prevention of SG, we conducted a study to determine the incidence of SG in primiparous women in our population and identify the risk factors associated with their development. In addition to previously studied risk factors, we looked at other factors, not studied in the past, that may theoretically affect the risk of developing SG such has smoking history and fetal gender.

## Materials and methods

A cross-sectional study was conducted at a large private teaching hospital in Beirut, Lebanon, after obtaining institutional review board approval. All primiparas with singleton gestations delivering during a 6-month period (February-July 2005) were invited to participate in the study irrespective of gestational age at delivery. Women were identified through the Delivery Suite logbook on a daily basis.

After obtaining written consent to participate in the study, all eligible participants were assessed during the postpartum period before their discharge from the hospital using a 22-item data collection tool. Information was collected from the medical charts about socioeconomic status, gestational age at delivery, total weight gain during pregnancy, current weight, fetal gender, and birthweight. Socioeconomic status was determined based on the third party coverage with patients admitted on the expense of the Ministry of Health labelled as low socioeconomic status and those who had private insurance as high socioeconomic status. Patients were also asked about the use of creams for prevention of SG during pregnancy, smoking history, and family history of stretch marks. Family history of SG was considered positive if the woman’s mother and/or sister had developed SG during her pregnancy. Skin type was determined by interview questions based on the Fitzpatrick classification, which is based on how often a person burns and how well they tan when exposed to the sun.^11^

Presence of SG on the abdomen, thighs, and breasts was assessed by 1 of 3 researchers based on a scale developed and validated by the research team. The scale is based on the total surface area of the affected body part that is covered by SG: < 25% was rated as mild, 25-50% as moderate, and > 50% as severe. The scale provided a useful way to incorporate the number of SG as well as the width of SG covering the affected area. In return for their participation, women were given a packet of brochures about the postpartum period, breast feeding, and newborn care.

Assuming a 50% prevalence of SG,^10^ a total of 113 patients would be required to achieve a clinical significance of 15% at a power of 90% and a significance level of .05. The data were entered and analyzed using SPSS 13.0 (Chicago, IL). Two different outcomes were considered: (1) women with any SG (mild, moderate, or severe) on either the abdomen, thighs, or breasts versus those with no SG in any of those sites; and (2) women with moderate and/or severe SG in any of the 3 sites versus women who had either mild SG or none. Comparing the 2 outcomes across women’s characteristics was done by either performing chi-square tests if the variables were categorical or *t* tests if the variables were continuous. Statistical significance was set at α = 0.05.

## Results

During the study period, 532 women delivered at the hospital. Of these, 163 were eligible for participation in the study. Forty-one women were discharged before it was possible to approach them, and 9 were not interested in participating in the study. One woman who was eligible was not approached because her infant was stillborn. Of the 112 women that were assessed, 1 had missing information for SG on the abdomen, thigh, and breast and another had no SG on abdomen and thigh but had missing information on the breast. Thus, both patients were excluded from the final analysis.

All eligible patients who were not formally assessed were compared to the women (n = 110) included in the study. No significant differences were found in maternal age, socioeconomic status, gestational age at delivery, fetal gender, or birthweight.

**FIGURE 1:**
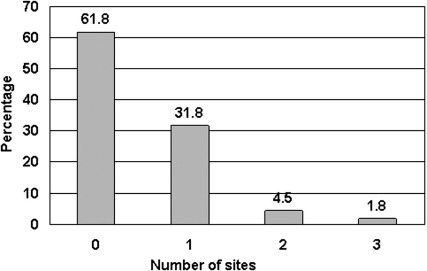
Proportion of women who developed striae gravidarum by number of anatomic sites involved

**FIGURE 2:**
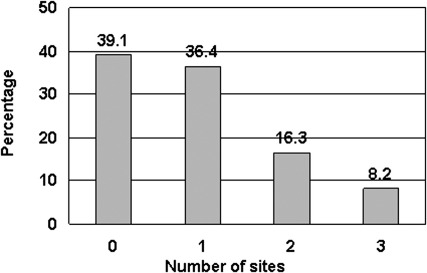
Proportion of women with moderate or severe striae gravidarum by number of anatomic sites involved

Of the 110 women enrolled in the study, 67 (61%) developed SG in at least 1 of the assessed sites. Fifty-three women (48%) developed SG on the abdomen, 27 (25%) developed SG on the breasts, and 27 (25%) developed SG on the thighs during their pregnancy. Figure 1 shows the percentage of women who developed SG at 1, 2, or all 3 sites. Of the abdominal striae, 17 (32%) were mild, 18 (34%) moderate, and another 18 (34%) severe. The severity of the striae on the breasts and thighs of the women were similar to each other with 19 (70%) reported as mild, 7 (26%) as moderate, and only 1 (4%) as severe. Figure 2 depicts the risk of developing moderate/severe SG by number of sites involved.

Most of the women in the study (93%) delivered at term. Seven percent delivered before 37 weeks of gestation with 1 woman delivering as early as 27 weeks. Eleven of the women (10%) delivered past 40 weeks of gestation. Maternal age ranged between 19 and 46 years. The majority of the women were between 24 and 34 years of age (77%), and the mean maternal age was 28 years. The weight gained during the pregnancy ranged from 3 to 33 kg with a mean of 14.4 kg. The BW ranged between 677 and 4115 g with a mean BW of 3143 g. Table 1 summarizes some antenatal and fetal characteristics in both groups. Women who developed SG were significantly younger and had gained significantly more weight during pregnancy compared to those who did not. BW and gestational age at delivery were strongly associated with risk of developing moderate/severe SG.

The predominant skin type in our population was Fitzpatrick III (41%) and IV (32%). Twenty percent of the women had skin types I/II, and only 8% had skin types V/VI. Most of the women (88%) were nonsmokers, and 45% were of a low socioeconomic status. Only 6% were current smokers and continued to smoke during their pregnancy, and 7% had smoked in the past but were no longer smoking at diagnosis of pregnancy. Sixty-seven women (61%) had used a cream or lotion during their pregnancy in an attempt to avoid the development of SG, and 19 (17%) had used more than 1 cream or lotion. There was a large variation in the types of creams used. The most commonly used products were cocoa butter (11%), baby oil (10%), and almond oil (5%). Sixty-five (59%) of the infants delivered were males and 47 (43%) of them were females. No relationship was noted between skin type, socioeconomic status, smoking, cream use, fetal gender, or family history and the risk of developing SG. However, women with family history of SG were more likely to develop moderate/severe SG than were those with no family history of SG. These findings are summarized in Table 2.

## Comment

This study provides a clinical assessment of the prevalence of SG and associated risk factors in a cohort of racially homogeneous women at a single tertiary-care referral center. The evaluation was based on a new scoring system that was developed by the researchers. This is 1 of the few studies in which SG were quantified by clinical assessment rather than relying on the woman’s own evaluation of her SG. To the best of our knowledge, our study is the only study that evaluated SG on the breasts and thighs and not merely the abdomen as in previously published studies in the literature. This was confirmed by a MEDLINE search from 1966 to March 2006, using the keywords “striae gravidarum,” “stretch mark,” and “pregnancy.” Our finding that 24% of women developed SG on their thighs or breasts shows that these areas are also significantly affected by SG.

We found that the prevalence of SG is 60%, consistent with previous reported figures.^1,5^ SG have a predilection to the abdomen, the site of involvement in 47% of the women; 24% had SG on the thighs and/or breasts. The correlation we identified between weight gain during pregnancy and BW and the development of SG are consistent with the findings by Davey.^4^ Although gestational age at delivery and BW were similar in those who developed SG and those who did not, both BW and gestational age at delivery were significantly larger for those who developed more severe SG. These factors are all probably interrelated and are related to some extent to the degree of stretching of the skin. Thomas et al noted that women with higher BMI and larger babies have more stretch marks.^5^

Similar to Thomas et al,^5^ we found that younger women were more likely to develop SG, though this finding is not consistent with other studies.^4^

Although we did not find that family history of SG was significantly correlated with the development of SG, we did find that women with a positive family history of SG were more likely to develop moderate/severe SG, suggesting that genetic factors do play a role in the development of SG. The other study that assessed the role of family history found a strong relationship between family history of SG and the risk of their development. However, that study was a voluntary and self-administered questionnaire that did not take into account factors like maternal age, number of births, and time elapsed since the delivery date.^1^

The literature on the association between race and risk of the development of SG is conflicting with some researchers finding that nonwhites were more likely to develop SG than white women,^1^ while others found that women with lighter skin were more likely to develop SG.^12^ Unfortunately, our study population was too racially homogeneous to determine any differences with regard to SG risk related to skin type.

We felt it would be worthwhile to investigate the effects of smoking and fetal gender, mostly due to the theoretical effect of tobacco and fetal sex hormones on SG development since both are known to affect connective tissue properties. However, the proportion of smokers in our cohort was too small to show any difference with regard to SG development. We found that fetal gender did not correlate with SG development.

A large proportion of our population was using 1 or more creams/lotions in an attempt to prevent the development of SG; however, we found no correlation between cream use and SG development. In 1972, Davey found that the use of oil and massage significantly reduced the likelihood of SG development.^4^ The nature of our study makes it difficult to draw any conclusions regarding a possible advantage or role of some of the creams utilized. It is very likely that users of lotions/creams started to apply these topical treatments after they noted that they were developing SG in the hope of minimizing their appearance.

The appearance of SG may be influenced by population genetics. If that is the case, the significance level (α) should probably be set to lower levels which may affect our results. Genetic studies to establish whether such a linkage exists have already been proposed to clarify this issue.^13^

Pregnant women often request information regarding their risks of developing SG and means to prevent their appearance during their prenatal visits. Our findings can help physicians answer some of these questions when counseling patients about their risk of developing SG. Although some of the factors associated with SG are not modifiable (ie, age and family history), other factors such as weight gain during pregnancy are. Future research should focus on preventive methods that may reduce the likelihood of SG development. More specifically the prophylactic use of creams and lotions should be further investigated to determine once and for all if these treatments have any benefit.

## Figures and Tables

**TABLE 1:** Distribution of striae gravidarum by age (maternal and gestational) and weight (maternal and fetal)

	Presence of SG			Moderate/Severe SG		
Yes (n = 67)	No (n = 43)	*P* value	Yes (n = 42)	No (n = 68)	*P* value
Maternal age (y)	26.5 ± 4.5	30.5 ± 4.6	< .001⁎	26.2 ± 4.1	29.2 ± 5.1	.002⁎
Gestational age (wk)	39.0 ± 1.8	38.4 ± 2.7	.202	39.4 ± 1.3	38.4 ± 2.5	.015⁎
Weight gained (kg)	15.6 ± 3.9	12.5 ± 4.5	< .001⁎	16.1 ± 3.9	13.3 ± 4.4	.001⁎
Birthweight (g)	3230 ± 46	3020 ± 59	.050	3300 ± 35	3040 ± 59	.010⁎

Data presented as mean ± standard deviation. SG, striae gravidarum.

⁎ Statistically significant.

**TABLE 2:** Distribution of striae gravidarum and degree of severity by selected maternal characteristics

	Presence of SG			Moderate/Severe SG		
Yes N (%)	No N (%)	*P* value	Yes N (%)	No N (%)	*P* value
Family history of SG						
Yes	48 (64.9)	26 (35.1)	.151	34 (45.3)	40 (54.1)	.019⁎
No	16 (50.0)	16 (50.0)		7 (21.9)	25 (78.1)	
Cream use						
Yes	43 (64.2)	24 (35.8)	.380	28 (41.8)	39 (58.2)	.331
No	24 (55.8)	19 (44.2)		14 (32.6)	29 (67.4)	
Current smoking†						
Yes	4 (57.1)	3 (42.9)	.779	3 (42.9)	4 (57.1)	.713
No	59 (62.1)	36 (37.8)		37 (38.9)	58 (61.1)	
Stopped	4 (50.0)	4 (50.0)		2 (25.0)	6 (75.0)	
Skin type-Fitzgerald classification						
I/II	12 (54.5)	10 (45.5)	.132	9 (40.9)	13 (59.1)	.923
III/IV	52 (65.8)	27 (34.2)		30 (38.0)	49 (62.0)	
V/VI	3 (33.3)	6 (66.7)		3 (33.3)	6 (66.7)	
Baby’s sex						
Male	38 (60.3)	25 (39.7)	.883	23 (36.5)	40 (63.5)	.676
Female	29 (61.7)	18 (38.3)		19 (40.4)	28 (59.6)	
Socioeconomic status						
High	34 (56.7)	26 (43.3)	.439	21 (35.0)	39 (65.0)	.699
Low	33 (66.0)	17 (34.0)		21 (42.0)	29 (58.0)	

*SG*, striae gravidarum.

⁎ Statistically significant.

† Current Smoking was labeled ‘yes’ if the patient smoked during pregnancy, ‘no’ if she has never smoked, and ‘stopped’ if smoking was stopped before pregnancy.
